# Single Nucleotide Polymorphisms in the Leptin-a Gene and Associations with Growth Traits in the Orange-Spotted Grouper (*Epinephelus coioides*)

**DOI:** 10.3390/ijms14048625

**Published:** 2013-04-22

**Authors:** Yun Wei, Hai Huang, Zining Meng, Yong Zhang, Jian Luo, Guohua Chen, Haoran Lin

**Affiliations:** 1 Key Laboratory of Tropical Biology Resources, Ministry of Education, College of Ocean, Hainan University, Haikou 570228, China; E-Mails: 15954809536@163.com (Y.W.); luojian@yahoo.cn (J.L.); chguh3240@yahoo.com.cn (G.C.); 2 Sanya Science & Technology Academy of Hainan National Breeding and Multiplication, Sanya 572000, China; 3 State Key Laboratory of Biocontrol, Institute of Aquatic Economic Animals and Guangdong Provincial Key Laboratory for Aquatic Economic Animals, College of Life Sciences, Sun Yat-Sen (Zhongshan) University, Guangzhou 510275, China; E-Mails: mengzn@mail.sysu.edu.cn (Z.M.); lsszy@mail.sysu.edu.cn (Y.Z.)

**Keywords:** leptin, single nucleotide polymorphisms (SNPs), molecular markers, orange-spotted grouper (*Epinephelus coioides*), growth traits, association analysis

## Abstract

Leptin is a multifunctional protein involved in processes such as body weight regulation, energy expenditure, fat metabolism, food intake, and appetite regulation. Duplicate leptin genes, leptin-a and leptin-b, were previously detected in the orange-spotted grouper. In this study, we cloned the full-length open reading frame (ORF) of the leptin-a gene in the orange-spotted grouper, searched for polymorphisms, and performed association analyses between these polymorphisms and seven growth traits. Six polymorphisms, consisting of 2 SNPs in intron 1 (c.182T > G, c.183G > T) and 4 SNPs in exon 2 (c.339C > G, c.345C > T, c.447G > A, c.531C > T), were identified and genotyped in 200 individuals. The c.182T > G and c.183G > T polymorphisms showed complete linkage and were analyzed together. Association analyses revealed that the c.182 + 183TG > GT polymorphism was significantly associated with body weight (BWT) and body width (BWH), with the AB (TG/GT) genotype showing positive effects on growth traits. Additionally, the SNP c.447G > A was significantly associated with BWT, BWH, overall length (OL), trunk width (TW), and head length (HL), with the GA genotype displaying positive effects on growth traits. The c.531C > T SNP showed a close association between the TT genotype and decreased growth. Our results demonstrate that several polymorphisms in the leptin-a gene are associated with growth traits and can be used for marker-assisted selection (MAS) in orange-spotted grouper populations.

## 1. Introduction

The orange-spotted grouper (*Epinephelus coioides*) belongs to the subfamily Epinephelinae (family, Serranidae) and mainly inhabits the Indian Ocean, the Red Sea, the Mediterranean Sea, the Northern Pacific Ocean, the Western Pacific Ocean and Southeast Asia [1,2]. It is a highly valued cultured marine species and is farmed in many countries including China, Thailand, Singapore, Japan, Indonesia, and Brunei Darussalam [3]. According to FAO fishery statistics, the global aquaculture production value of the orange-spotted grouper has increased by almost 100-fold from 1999 to 2009, from 33,000 to 2,354,000 US dollars [4]. Currently in China, the orange-spotted grouper has become a major food fish in live fish markets and is an important cultured fish for commercial sale in the Guangdong, Hainan, Taiwan and Fujian provinces. The vast market demands for orange-spotted grouper have driven efforts to breed families and populations with higher growth rates and lower food coefficients. The application of marker-assisted selection (MAS) to the orange-spotted grouper is a promising strategy for improving growth traits, and the identification of genetic markers associated with growth traits could contribute to genetic improvements in cultured fishes and advance the MAS process.

Leptin, a product of the obese (ob) gene first discovered in the mouse by Zhang *et al*. in 1994 [5], is a member of the class-I helical cytokine family. In mammals, leptin has been demonstrated to perform important roles in body weight regulation, energy expenditure, fat metabolism, food intake, appetite regulation, bone remodeling, hematopoiesis, immune function and reproduction [6–8]. In teleosts, its function remains unclear, although several studies have been conducted on the teleost leptin genes, which was first cloned in the pufferfish (*Takifugu rubripes*) [9]. Since then, and in keeping with the genome duplication observed in teleosts, leptin genes have been identified in several teleost species, including the common carp (*Cyprinus carpio*) [10], zebrafish (*Danio rerio*) [11], Japanese medaka (*Oryzias latipes*) [12], grass carp (*Ctenopharyngodon idella*) [13], rainbow trout (*Oncorhynchus mykiss*) [14], Atlantic salmon (*Salmo salar*) [15], striped bass (*Morone saxatilis*) [16], goldfish (*Carassius auratus*) [17] and orange-spotted grouper [18]. Unlike other vertebrate animals, which have only a single copy of the leptin gene, duplicate leptin genes have been identified in some teleost fish, such as zebrafish [11], Japanese medaka [12], and orange-spotted grouper [18]. With respect to the cloned leptin genes in fish, leptin a type was found to be present in the majority of species studied [19]. In the orange-spotted grouper, leptin-a mRNA was found to be expressed in most tissues at generally higher levels than that of leptin-b. The highest expression of leptin-a was found in cerebellum, liver and ovary, which suggests that the expression protein of leptin-a gene may have pleiotropic physiological effects [18]. Compared to the leptin-b gene, the leptin-a gene is more likely to affect growth traits in the orange-spotted grouper.

Several polymorphisms of the leptin genes have been associated with economically relevant traits in many farmed animals, especially swine [20] and cattle [21–23]. However, in teleost fish, no papers have reported polymorphisms in the leptin genes and their associations with growth traits.

The objectives of this study were to identify single nucleotide polymorphisms (SNPs) in the leptin-a gene and to analyze associations between these polymorphisms and growth traits in the orange-spotted grouper. In teleost fish, this is the first study of leptin polymorphisms and their associations with growth traits, and these findings will help to evaluate the use of leptin genes in marker-assisted selection (MAS) of the orange-spotted grouper and other cultured fish.

## 2. Results and Discussion

### 2.1. SNP Identification and Genotyping

The open reading frame (ORF) of the leptin-a gene in the orange-spotted grouper contains 2 exons of 152 bp and 334 bp, which code for a 161-amino acid protein. One intron of 87 bp was identified between the 2 exons. Six SNPs were identified by alignment of leptin-a gene fragments from 200 individuals. Two mutations were found in intron 1 (c.182T > G, c.183G > T), and 4 synonymous mutations were found in exon 2 (c.339C > G, c.345C > T, c.447G > A, c.531C > T). The observed and expected heterozygosity ranged from 0.030 to 0.205 and from 0.030 to 0.251, respectively. One locus (c.531C > T) deviated significantly from Hardy-Weinberg equilibrium (*p* < 0.05). All the 6 SNPs presented low polymorphisms (PIC < 0.25). In the experimental population, heterozygous mutational genotypes were detected for the 6 SNPs, but recessive homozygosis mutational genotype was only detected for the c.531C > T SNP (Table 1).

**Figure 1:**
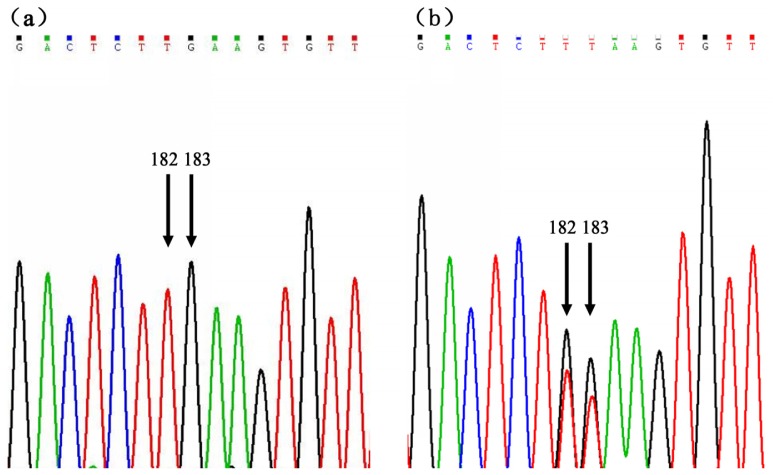
Chromatogram files of the SNPs c.182T > G and c.183G > T for the (**a**) dominant homozygotic genotype and (**b**) recessive heterozygous genotype.

Our results showed that the 2 mutations in intron 1 (c.182T > G, c.183G > T) appeared in pairs and were in complete linkage. All of the fragments from individuals with the mutational genotype presented the same chromatograms, which are shown in Figure 1b. Using comparative analysis of chromatograms from individuals with normal and mutational genotypes (Figure 1), we deduced that these SNPs (c.182T > G, c.183G > T) were produced by base exchange between the T of locus 182 and the G of locus 183. Therefore, in association analyses, these 2 SNPs (c.182T > G, c.183G > T) were analyzed together and were marked as a single locus (c.182 + 183TG > GT). For the locus (c.182 + 183TG > GT), AA represents the dominant homozygous normal genotype (TT/GG) and AB represents the recessive heterozygous mutational genotype (TG/GT).

### 2.2. Association Analysis with Growth Traits

To investigate the effects of leptin-a SNPs, association analyses between SNP genotypes and seven growth traits were performed. The results are shown in Table 2.

**Figure 2:**
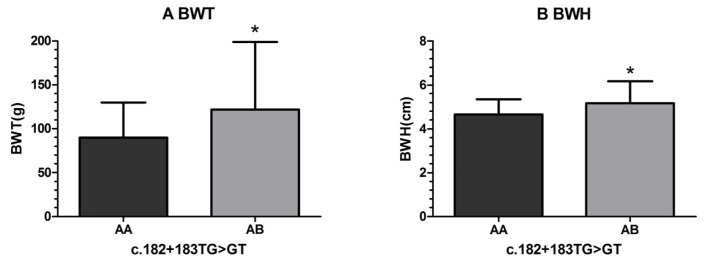
Significant differences in growth traits observed between different genotypes of c.182 + 183TG > GT.

For the mutational locus c.182 + 183TG > GT, the body weight (BWT) and body width (BWH) measurements of fish with the AB (TG/GT) genotype were significantly greater than those with the AA (TT/GG) genotype (Figure 2A,B); the overall length (OL), trunk width (TW), head length (HL), caudal peduncle length (CPL), and condition factor (K) of fish with the AB (TG/GT) genotype were also greater than those of fish with the AA (TT/GG) genotype, but the differences were not significant. These results indicated that the mutation c.182 + 183TG > GT were significantly associated with growth traits, with the heterozygous mutation genotype AB (TG/GT) having positive effects on growth traits.

The c.339C > G polymorphism did not show a significant association with any of the growth traits. For the c.345C > T SNP, the K value of individuals with the CT genotype was significantly lower than that of those with CC genotype, but individuals with the CT genotype displayed inconsistent divergences in other traits.

**Figure 3:**
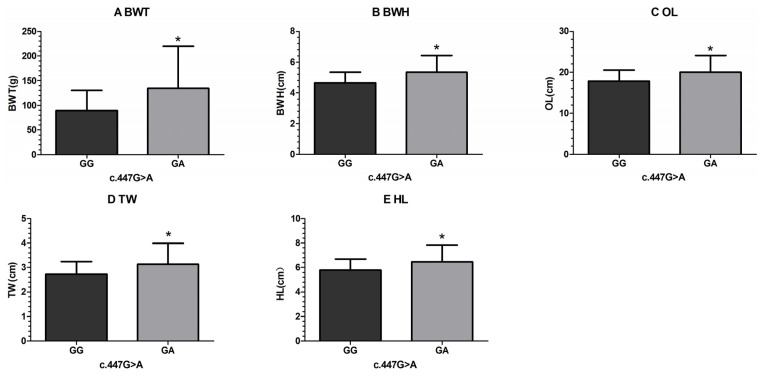
Significant differences in growth traits between observed different genotypes of c.447G > A.

For the c.447G > A SNP, the BWT, BWH, OL, TW and HL of fish with the GA genotype were significantly greater than those of fish with the GG genotype (Figure 3A–E); fish with the GA genotype also showed higher CPL and K values. These results indicated that the c.447G > A SNP is significantly associated with growth traits, with the heterozygous mutation genotype GA having positive effects on growth traits.

For the c.531C > T SNP, there were no significant differences between the CC and CT genotypes for any of the growth traits; the BWT, BWH, OL, TW, HL and CPL of fish with the TT genotype were much lower than those with the CC and CT genotypes, but these differences were not significant. These results indicated that the TT genotype has negative effects on growth traits and that the c.531C > T SNP is likely associated with growth traits.

### 2.3. Discussion

Our results revealed that the mutation c.182 + 183TG > GT is highly associated with growth traits of the orange-spotted grouper and that the AB (TG/GT) genotype positively affects such growth traits. The mutation c.182 + 183TG > GT is a rare and novel polymorphism with complete linkage between SNPs c.182T > G and c.183G > T. If the genotypes of two or more SNPs were linked, it would be informative and have wide application for MAS. Our results concerning the locus studied (c.182 + 183TG > GT) demonstrate that a polymorphism within the intron of this gene is associated with growth traits in the orange-spotted grouper. Introns span a much larger portion of the genome than exons [24], and introns are usually more highly diversified than adjacent exons [25]; as a result, more polymorphisms can be identified in introns. Introns are not involved in transcription, but they have proven to be regulatory elements of protein expression [26], and the polymorphism in intron has been found to be associated with growth traits in the chicken growth hormone (GH) gene [27]. In light of recent achievements in genome sequencing and the development of high-throughput sequencing, detecting and identifying polymorphisms in introns should become much easier, and the development of molecular markers associated with growth traits in introns therefore holds great promise.

The SNP c.447G > A was found to be significantly associated with growth traits, with the GA genotype positively affecting such growth traits. Furthermore, the SNP c.531C > T is a polymorphism likely associated with growth traits, with the TT genotype having negative effects on growth traits. These 2 SNPs were synonymous mutations in exon 2, and 2 hypotheses could explain how synonymous mutations may affect growth traits: (1) synonymous mutations may indirectly affect gene functions by affecting alternative splicing, splicing efficiency, messenger RNA turnover, and subsequent gene expression; or (2) synonymous mutations may be in linkage disequilibrium (LD) with one or more nearby quantitative trait loci (QTL) associated with growth traits [28]. Previous studies have reported synonymous mutations affecting growth traits. For example, Zhang (2006) identified a synonymous mutation, T123G, at 123 bp of 26th exon in the chicken apolipoprotein B (apoB) gene affecting body growth and fatness traits [29], and Sun *et al*. (2012) found that 2 synonymous mutations in exon 3 of the myostatin (MSTN) gene positively impact the growth traits in the common carp [28].

Several studies in animal husbandry have also reported that SNPs in leptin genes are associated with growth traits. Stachowiak *et al*. (2007) investigated SNPs associated with fatness traits in the porcine leptin gene promoter [30], and Kulig *et al*. (2009) reported that the leptin SNP A59V polymorphism significantly affected the body weight and the average daily weight gain in Limousin cattle [31]. Gill *et al*. (2009) found that the leptin gene SNP UASMS2 significantly affected meat quality traits of Aberdeen Angus-cross beef cattle, as assessed by a taste panel [32], and Clempson *et al*. (2011) reported 4 leptin SNPs (A1457G, A59V, UASMS1, UASMS2) that were associated with different types of growth performance in Holstein cows [33]. Melucci *et al*. (2012) found that beef quality traits of cows were affected by a SNP in exon 2 of the leptin gene (E2FB) [34], and polymorphisms of the leptin receptor genes have been shown to be associated with growth traits in swine [35], chicken [36] and cattle [37]. In conclusion, these reports have revealed leptin genes to be important candidate gene groups associated with growth traits. However, no studies have reported polymorphisms in the leptin genes associated with growth traits in cultured fish or teleost fish, and our work is therefore the first to investigate this area of research.

Our study clearly demonstrates that the AB (TG/GT) genotype of c.182 + 183TG > GT, the GA genotype of c.447G > A and the TT genotype of c.531C > T display concerted effects on growth traits in the orange-spotted grouper. Therefore, the polymorphisms c.182 + 183TG > GT, c.447G > A, and c.531C > T in the leptin-a gene could be candidates for development of molecular markers associated with growth traits in this species.

## 3. Experimental Section

### 3.1. Materials and Phenotypic Data Collection

The experimental population was generated by the parent fish, 29 males and 12 females, which were the first generation progeny of a wild broodstock collected from the South China Sea near Hainan Island. On 10 December 2010, approximately 1 month after hatching, 15,000 fry were transported and placed into one net cage built in the fisheries area in Haitang bay of Sanya (Hainan Province, China) on the South China Sea. The experimental stock was maintained under constant environmental conditions and was fed according to the management practices of the fishery farm. On 8 August 2011, 200 juvenile fishes were sampled randomly from the cage. These samples were assigned numbers from 1 to 200. Eleven phenotypic traits were measured and recorded, including BWT, BWH, OL, body length (BL), TW, HL, caudal peduncle width (CPW), CPL, snout length (SL), eyeball diameter (ED), and interorbital distance (ID). We also calculated K as a growth trait according to the following formula: *K* = 100 BWT/BL^3^. Descriptive statistics of the growth traits observed are presented in Table 3. Tissues were collected from fin clips and were preserved in 95% ethanol at −20 °C until processing for DNA isolation. Total genomic DNA extractions from fin clips were performed according to the proteinase K/phenol extraction protocol previously described by Sambrook and Russell [38]. The concentration of genomic DNA was determined by agarose gel electrophoresis using a UV spectrophotometer.

### 3.2. PCR Amplification and SNP Identification

To detect leptin-a polymorphisms, we determined the nucleotide coding sequences of the leptin-a gene from genomic DNA. Two pairs of primers were designed based on *Epinephelus coioides* leptin-a mRNA information (GenBank accession No.JX406147.1) using Primer Premier 5 software (PREMIER Biosoft, Palo Alto, CA, USA). Nucleotide sequences of the primers used are shown in Table 4.

PCR amplifications were conducted in a final volume of 50 μL, containing 5 μL 10× LA PCR Buffer II (Mg^2+^ Plus), 5 μL dNTPs (2.5 mM each), 1 μL of each primer (20 μM), 0.5 μL of LA Taq DNA polymerase (5 U/μL, TaKaRa, Dalian, China) and 1 μL of genomic DNA (50–100 ng/μL). The PCR amplification reactions were performed in an ABI 2720 thermal cycler (Applied Biosystem, Foster City, CA, USA), with the following thermal cycling conditions: denaturation at 95 °C for 3 min; 40 cycles of amplification for 30 s at 95 °C, 1 min at 55 °C, and 1 min at 72 °C; and a final extension at 72 °C for 10 min. Amplification results were verified by 1.5% agarose gel electrophoresis. PCR fragments of the predicted size were cut and purified from gels with an agarose gel DNA Extraction kit (D823A, TaKaRa, Dalian, China).

### 3.3. SNP Identification and Genotyping

Fragments were sequenced by direct sequencing using an ABI 3730 DNA Analyzer (Applied Biosystems, Foster City, CA, USA). Sequence mutations differing between individuals were detected using the ClustalX Multiple Sequence Alignment Program, version 1.83 [39]. SNPs were identified and genotyped by analyzing and comparing chromatogram files using Chromas version 2.33 (Technelysium Pty Ltd., South Brisbane, Australia) and SeqMan Pro version 7.1.0(44.1) in the DNASTAR lasergene core suite software (DNASTAR Inc., Madison, WI, USA).

One pooled sample mixed with leptin-a gene DNA fragments of 42 individuals was sequenced based on Ion Torrent DNA high-throughput sequencing platform (Life Technologies, South San Francisco, CA, USA). The SNPs of the leptin-a gene were verified by comparing the results of high-throughput sequencing and direct sequencing.

### 3.4. Statistical Analysis

Allele and genotype frequencies were calculated using a simple allele counting method. Hardy-Weinberg equilibrium was tested for goodness-of-fit by comparing expected and observed genotype frequencies using a chi-square test.

The 12 growth traits were synthetically evaluated to determine the universality, the standard deviation, the coefficient of variance, and the degree of normal distribution (Table 3). Seven traits, including BWT, BWH, OL, TW, HL, CPL and K, were selected as representative growth traits of the orange-spotted grouper.

Association analyses between genotypes of leptin-a and 7 growth traits were performed using general linear model (GLM) procedure with SPSS 20.0 software (IBM, Armonk, NY, USA). We used the following statistical model: *Y* = *u* + *G* + *e*, where *Y* is the phenotypic value of each trait; u is population mean value of 7 growth traits, G is the fixed genotype effect of each SNP, and e is the random error effect. Multiple comparisons between different genotypes were tested using the LSD method with Bonferroni correction adjustment.

## 4. Conclusions

We obtained the gene sequence and identified 6 SNPs in the leptin-a gene of the orange-spotted grouper. Association analysis with seven growth traits revealed that: 2 SNPs in intron 1 (c.182T > G, c.183G > T) were in complete linkage and were significantly associated with BWT and BWH, with the mutation genotype AB (TG/GT) showing positive effects on such growth traits. Additionally, the SNP c.447G > A was significantly associated with BWT, BWH, OL, TW, and HL, with the genotype GA positively affecting these growth traits. Finally, the SNP c.531C > T in exon 2 was associated with growth traits, and fish with the genotype TT showed poorer growth performance. This is the first study in teleost to provide further evidence for the association of leptin gene polymorphisms with growth traits, and these findings could lead to a greater understanding of the functions and regulation of leptin gene expressions related to fish growth. These results also validate to the candidate gene approach and the use of the leptin-a gene for MAS in the orange-spotted grouper.

## Figures and Tables

**Table 1:** SNPs in the orange-spotted grouper leptin-a gene: genotype and allele frequencies, polymorphism information content, and chi-square tests of goodness-of-fit for Hardy-Weinberg equilibrium law in the experimental population.

SNP	Position	Mutation type	Sample size	Genotype frequencies (%)			Allele frequencies (%)		*H*o	*H*e	*p*-value (χ^2^, HWE)	PIC a
**c.182T > G**	Intron1	Untranslated	200	TT	TG	GG	T	G	0.055	0.053	0.923	0.052
				94.5	5.5	0	97.25	2.75				
**c.183G > T**	Intron1	Untranslated	200	GG	GT	TT	G	T	0.055	0.053	0.923	0.052
				94.5	5.5	0	97.25	2.75				
**c.339C > G**	Exon2	Synonymous	200	CC	CG	GG	C	G	0.030	0.030	0.977	0.029
		T:ACC→ACG		97.0	3.0	0	98.50	1.50				
**c.345C > T**	Exon2	Synonymous	200	CC	CT	TT	C	T	0.075	0.072	0.859	0.070
		N:AAC→AAT		92.5	7.5	0	96.25	3.75				
**c.447G > A**	Exon2	Synonymous	200	GG	GA	AA	G	A	0.040	0.039	0.959	0.038
		P:CCG→CCA		96.0	4.0	0	98.00	2.00				
**c.531C > T**	Exon2	Synonymous	200	CC	CT	TT	C	T	0.205	0.251	0.033*	0.220
		L:CTC→CTT		75.0	20.5	4.5	85.25	14.75				

a PIC = polymorphism information content; loci present high polymorphisms (PIC > 0.5); loci present moderate polymorphisms (0.25 < PIC < 0.5); loci present low polymorphisms (PIC < 0.25);

* Significant at the *p* < 0.05 level.

**Table 2:** Least squares means and standard deviations of growth traits between different genotypes of leptin-a SNPs in the Orange-spotted grouper.

SNP	Genotypes	*N*	BWT (g)	BWH (cm)	OL (cm)	TW (cm)	HL (cm)	CPL (cm)	*K* (%)
c.182 + 183	AA(TT/GG)	189	89.67 ± 3.12 ^a^	4.65 ± 0.05 ^a^	17.89 ± 0.20	2.73 ± 0.04	5.79 ± 0.07	2.44 ± 0.04	2.67 ± 0.32
TG > GT	AB(TG/GT)	11	121.82 ± 12.94 ^b^	5.17 ± 0.22 ^b^	19.32 ± 0.82	3.03 ± 0.16	6.23 ± 0.28	2.66 ± 0.15	2.74 ± 0.38
	*p*-value		0.017*	0.020*	0.091	0.074	0.130	0.160	0.518
c.339C > G	CC	194	91.26 ± 3.12	4.68 ± 0.05	17.97 ± 0.20	2.75 ± 0.04	5.82 ± 0.07	2.46 ± 0.04	2.67 ± 0.32
	CG	6	97.33 ± 17.77	4.83 ± 0.30	18.02 ± 1.12	2.83 ± 0.22	5.83 ± 0.38	2.45 ± 0.21	2.81 ± 0.34
	*p*-value		0.737	0.602	0.964	0.692	0.963	0.980	0.285
c.345C > T	CC	185	90.51 ± 3.19	4.67 ± 0.05	17.90 ± 0.20	2.74 ± 0.04	5.80 ± 0.07	2.45 ± 0.04	2.69 ± 0.32 ^a^
	CT	15	102.93 ± 11.21	4.87 ± 0.19	18.77 ± 0.70	2.83 ± 0.14	6.03 ± 0.24	2.49 ± 0.13	2.51 ± 0.27 ^b^
	*p*-value		0.288	0.303	0.234	0.520	0.346	0.802	0.036*
c.447G > A	GG	192	89.64 ± 3.08 ^a^	4.65 ± 0.05 ^a^	17.88 ± 0.20 ^a^	2.73± 0.04 ^a^	5.79 ± 0.07	2.44 ± 0.04	2.67 ± 0.02
	GA	8	134.75± 15.07 ^b^	5.34 ± 0.25 ^b^	20.01 ± 0.95 ^b^	3.15± 0.19 ^b^	6.48 ± 0.33	2.75 ± 0.18	2.72 ± 0.11
	*p-value*		0.004*	0.009*	0.030*	0.029*	0.040*	0.092	0.681
c.531C > T	CC	150	92.59 ± 3.55	4.70 ± 0.06	18.11 ± 0.22	2.76 ± 0.04	5.83 ± 0.08	2.48 ± 0.04	2.66 ± 0.30
	CT	41	91.66 ± 6.78	4.71 ± 0.11	17.80 ± 0.42	2.80 ± 0.08	5.88 ± 0.14	2.41 ± 0.08	2.72 ± 0.38
	TT	9	71.33 ± 14.47	4.30 ± 0.24	16.39 ± 0.90	2.38 ± 0.18	5.32 ± 0.31	2.31 ± 0.17	2.70 ± 0.32
	*p*-value		0.363	0.273	0.170	0.092	0.251	0.507	0.629
	CC/CT		1.000	1.000	1.000	1.000	1.000	1.000	1.000
	CT/TT		0.615	0.372	0.471	0.094	0.311	1.000	1.000
	TT/CC		0.466	0.343	0.200	0.116	0.335	1.000	1.000

**BWT** = body weight, **BWH** = body width, **OL** = overall length, **TW** = trunk width, **HL** = head length, **CPL** = caudal peduncle length, **K** = condition factor = 100 BWH/BL^3^, BL = body length;

* Significant at the *p* < 0.05 level;

a,b Multiple comparisons were performed using the least significant difference (LSD) test after Bonferroni correction adjustment; significant differences are shown using different superscripts (a *vs.* b) across the row; different superscripts within columns differ significantly (*p* < 0.05).

**Table 3:** Descriptive statistics and *p*-values from tests of normality for 12 growth traits. (*N* = 200).

Phenotypic traits a	Mean	Std. Error	Std. Deviation	CV b (%)	Minimum	Maximum	Range	*p*-value c (K-S test)
BWT (g)	91.440	3.070	43.418	47.48	20.00	310.00	290.00	0.000
BWH (cm)	4.681	0.051	0.726	15.51	2.70	7.50	4.80	0.076 *
OL (cm)	17.967	0.193	2.723	15.16	12.20	26.30	14.10	0.057 *
BL (cm)	14.774	0.167	2.368	16.03	10.00	22.10	12.10	0.041
TW (cm)	2.748	0.038	0.533	19.40	1.50	4.90	3.40	0.026
HL (cm)	5.816	0.065	0.925	15.91	3.80	9.00	5.20	0.000
CPW (cm)	1.606	0.017	0.243	15.13	1.10	2.50	1.40	0.000
CPL (cm)	2.455	0.036	0.506	20.59	1.30	3.80	2.50	0.000
SL (cm)	1.186	0.016	0.230	19.39	0.70	2.20	1.50	0.000
ED (cm)	0.946	0.007	0.093	9.84	0.60	1.20	0.60	0.000
ID (cm)	0.937	0.011	0.161	17.19	0.60	1.50	0.90	0.000
*K* (%)	2.676	0.023	0.320	11.96	1.78	3.53	1.75	0.200 *

a BWT = body weight, BWH = body width, OL = overall length, BL = body length, TW = trunk width, HL = head length, CPW = caudal peduncle width, CPL = caudal peduncle length, SL = snout length, ED = eyeball diameter, ID = interorbital distance, *K* = condition factor;

b CV = coefficient of variance, which was obtained by computing the ratio of the biased standard deviation to the mean;

c *p*-value is obtained by using Kolmogorov-Smirnov test with Lilliefors significance correction;

* At the *p* > 0.05 level; *p* > 0.05 means that the growth traits fit the normal distribution.

**Table 4:** Nucleotide sequences used in PCR assays for the leptin-a gene of the orange-spotted grouper (*Epinephelus coioides*).

Primer code	Primer sequences (5′–3′)	Product size	TM (°C)
**Leptin-a1**	F: ATGGACTACACTCTGGCCCTG	573 bp	51.0
	R: TCAGCAAGTCTCAAGATGGTCC		51.5
**Leptin-a2**	F: GGAACTACAGAACTACTTGGAA	698 bp	51.0
	R: GTGCTGGAGGAAATGTATTC		52.0
